# Supplementary material for: Development of Clinical-Grade Durvalumab-680LT and Nivolumab-800CW for Multispectral Fluorescent Imaging of the PD-1/PD-L1 Axis of the Immune Checkpoint Pathway
Source: Pharmaceuticals (Basel). 2025 Oct 7;18(10):1501. doi: 10.3390/ph18101501 (PMC12566847; doi:10.3390/ph18101501)

Supplementary

Table S1: Composition of different buffers for the buffer panel of durvalumab-680LT and nivolumab-800CW. API: Active Pharmaceutical Ingredient.

Component	Function	Concentration
Imfinzi® original formulation (pH 6.0)		
Durvalumab	API	50 mg/ml
Histidine/Histidine-HCl	Buffer	275 mM
Trehalose dihydrate	Isotonic agent	275 mM
Polysorbate 80	Protein stabilizer	0.02 mg/ml
Opdivo® original formulation (pH 6.0)		
Nivolumab	API	10 mg/ml
Sodiumchloride	Isotonicity agent	2.92 mg/mL
Sodium citrate dihydrate	Isotonic agent	5.88 mg/mL
Polysorbate 80	Protein stabilizer	0.02 mg/ml
Buffer 1 (pH 6.0)		
Durvalumab/nivolumab	API	1 mg/ml
Disodium monohydrogenphosphate	Buffer	0.534 mg/ml
Sodium dihydrogenphosphate	Buffer	7.332 mg/ml
Trehalose dihydrate	Isotonicity agent	74.0 mg/ml
Polysorbate 80	Protein stabilizer	0.02 mg/ml
Buffer 2 (pH 7.0)		
Durvalumab/nivolumab	API	1 mg/ml
Disodium monohydrogenphosphate	Buffer	5.46 mg/ml
Sodium dihydrogenphosphate	Buffer	3.02 mg/ml
Trehalose dihydrate	Isotonicity agent	74.0 mg/ml
Polysorbate 80	Protein stabilizer	0.02 mg/ml
Buffer 3 (pH 6.0)		
Durvalumab/nivolumab	API	1 mg/ml
Disodium monohydrogen phosphate	Buffer	0.534 mg/ml
Sodium dihydrogen phosphate	Buffer	7.332 mg/ml
Sodium chloride	Isotonicity agent	0.54 mg/ml
Polysorbate 80	Protein stabilizer	0.02 mg/ml
Buffer 4 (pH 7.0)		
Durvalumab/nivolumab	API	1 mg/ml
Disodium monohydrogen phosphate	Buffer	5.46 mg/ml
Sodium dihydrogen phosphate	Buffer	3.02 mg/ml
Sodiumchloride	Isotonicity agent	0.54 mg/ml
Polysorbate 80	Protein stabilizer	0.02 mg/ml
Buffer 5 (pH 6.0)		
Durvalumab/nivolumab	API	1 mg/ml
Disodium monohydrogen phosphate	Buffer	0.534 mg/ml
Sodium dihydrogen phosphate	Buffer	7.332 mg/ml
Sodiumchloride	Isotonicity agent	0.55 mg/ml
Buffer 6 (pH 7.0)		
Durvalumab/nivolumab	API	1 mg/ml
Disodium monohydrogen phosphate	Buffer	5.46 mg/ml
Sodium dihydrogen phosphate	Buffer	3.02 mg/ml
Sodiumchloride	Isotonicity agent	0.55 mg/ml
Buffer 7 (pH 6.0)		
Durvalumab/nivolumab	API	1 mg/ml

Disodium monohydrogen phosphate	Buffer	0.534 mg/ml
Sodium dihydrogen phosphate	Buffer	7.332 mg/ml
Trehalose dihydrate	Isotonicity agent	74.0 mg/ml
Buffer 8 (pH 7.0)		
Durvalumab/nivolumab	API	1 mg/ml
Disodium monohydrogen phosphate	Buffer	5.46 mg/ml
Sodium dihydrogen phosphate	Buffer	3.02 mg/ml
Trehalose dihydrate	Isotonicity agent	74.0 mg/ml

T*able S2: Label ratio results for durvalumab-680LT.*

	Conjugated product		Purified product			
Ratio	Monomer Integrity	Label efficiency	Monomer Integrity	Monomer concentration	Protein aggregates	Unconjugated IRDye
1:1	Pass	87.8%	Pass	Sufficient	N.D.	0.5%
2:1	Pass	88.4%	Pass	Sufficient	N.D.	0.5%
3:1	Pass	85.6%	Pass	Sufficient	N.D.	1.8%
4:1	Pass	86.1%	Pass	Sufficient	0.3%	2.1 %
8:1	Pass	82.3%	Pass	Sufficient	0.2%	3.9%
16:1	Pass	67.3%	Pass	Sufficient	0.9%	9.6%

N.D. Denotes 'Not Detected'

Table S3: Label ratio results for nivolumab-800CW.

	Conjugated product		Purified product			
Ratio	Monomer Integrity	Label efficiency	Monomer Integrity	Monomer concentration	Protein aggregates	Unconjugated IRDye
1:1	Pass	86.4%	Pass	Sufficient	N.D.	N.D.
2:1	Pass	89.9%	Pass	Sufficient	N.D.	0.3%
3:1	Pass	81.4%	Pass	Sufficient	N.D.	0.3%
4:1	Pass	79.3%	Pass	Sufficient	N.D.	0.4%
8:1	Pass	72.2%	Pass	Sufficient	N.D.	0.9%
16:1	Pass	63.8%	Pass	Sufficient	0.5%	2.1%

N.D. Denotes 'Not Detected'

Table S4: Stability results of durvalumab-680LT at storage for 12 months at 2-8 °C.

Test	Specification	0	1	3	6	12
Protein monomer concentration	Release: 0.95 – 1.05 mg/mL Shelf-life: 0.90 – 1.10 mg/mL	1.03 ± 0.01 mg/mL	1.02 ± 0.01 mg/mL	1.09 ± 0.006 mg/mL	1.01 ± 0.01 mg/mL	1.07 ±0.01 mg/mL
Protein aggregates	Release: ≤5.0% Shelf-life: ≤10%	N.D.	N.D.	N.D.	N.D.	N.D.
Unconjugated IRDye 680LT	Release: ≤5.0% Shelf-life: ≤10%	1.3 ± 0.1%	1.5 ± 0.06%	1.5 ± 0%	1.9 ± 0.1%	1.7 ± 0.06%
Protein monomer identity	Retention time comparable to reference standard	Conform	Conform	Conform	Conform	Conform
Protein monomer integrity	Peak shape comparable to reference standard; no shoulders, minimal tailing	Conform	Conform	Conform	Conform	Conform
Target binding affinity	EC_{50}ref/EC_{50}tracer 50 – 200%	15.466/15.83 97.7%	14.84/17.592 84.4%	10.97/15.669 70.0%	14.715/19.771 74.4%	14.366/21.476 66.9%
Appearance (turbidity)	Clear to slightly opalescent solution	Conform	Conform	Conform	Conform	Conform
Appearance (colour)	For information	Conform	Conform	Conform	Conform	Conform
Container closure and label	Closure intact, label legible and intact.	Conform	Conform	Conform	Conform	Conform
Extractable volume	≥5.0 mL	≥5.0 mL	≥5.0 mL	≥5.0 mL	≥5.0 mL	≥5.0 mL
pH	6.9 – 7.1	6.9	6.9	6.9	6.9	7.0
Osmolality	270 -310 mOsmol/kg	294 mOsmol/kg	291 mOsmol/kg	291 mOsmol/kg	293 mOsmol/kg	293 mOsmol/kg
Residual solvents (DMSO)	≤50.0 mg/L	16 mg/L	N.T.	N.T.	N.T.	N.T.
Bacterial endotoxins	≤5.0 EU/mL	<0.1 EU/mL	<0.1 EU/mL	<0.1 EU/mL	<0.5 EU/mL	<0.1 EU/mL
Sterility	Sterile	Sterile	N.T.	N.T.	Sterile	Sterile
UV-VIS absorption peaks	Peaks at 280 ± 3 nm and 679 ± 3 nm.	Conform	Conform	Conform	Conform	Conform
Visible particles	Practically free of particles	Conform	Conform	Conform	Conform	Conform
Subvisible particles	Particles ≥10 µm ≤6000/container Particles ≥25 µm ≤600/container	≥10 µm: 85 ≥25 µm: 3	N.T.	N.T.	N.T.	≥10 µm: 1239 ≥ 25 µm: 13

Table S5: Stability results of nivolumab-800CW at storage for 12 months at 2-8 °C.

Test	Specification	0	1	3	6	12
Protein monomer concentration	Release: 0.95 – 1.05 mg/mL Shelf-life: 0.90 – 1.10 mg/mL	0.98 ± 0.003 mg/mL	1.00 ± 0.01 mg/mL	1.01 ± 0.006 mg/mL	0.97 ± 0.003 mg/mL	0.97 ± 0 mg/mL
Protein aggregates	Release: ≤5.0% Shelf-life: ≤10%	N.D.	N.D.	N.D.	N.D.	0.7 ± 0.15%
Unconjugated IRDye 800CW	Release: ≤5.0% Shelf-life: ≤10%	0.4 ± 0.09%	N.D.	1.3 ± 0.03%	1.3 ± 0.05%	2.6 ± 0.15%
Protein monomer identity	Retention time comparable to reference standard	Conform	Conform	Conform	Conform	Conform
Protein monomer integrity	Peak shape comparable to reference standard; no shoulders, minimal tailing	Conform	Conform	Conform	Conform	Conform
Target binding affinity	EC_{50}ref/EC_{50}tracer 50–200%	42.554/70.709 60%	40.097/45.712 88%	37.343/59.823 64%	69.634/78.472 89%	39.577/43.021 92%
Appearance (turbidity)	Clear to slightly opalescent solution	Conform	Conform	Conform	Conform	Conform
Appearance (colour)	For information	Conform	Conform	Conform	Conform	Conform
Container closure and label	Closure intact, label legible and intact.	Conform	Conform	Conform	Conform	Conform
Extractable volume	≥5.0 mL	≥5.0 mL	≥5.0 mL	≥5.0 mL	≥5.0 mL	≥5.0 mL
pH	6.9 – 7.1	6.9	6.9	6.9	6.9	6.9
Osmolality	270 -310 mOsmol/kg	291 mOsmol/kg	290 mOsmol/kg	290 mOsmol/kg	290 mOsmol/kg	290 mOsmol/kg
Residual solvents (DMSO)	≤50.0 mg/L	28 mg/L	N.T.	N.T.	N.T.	N.T.
Bacterial endotoxins	≤5.0 EU/mL	<0.05 EU/mL	<0.05 EU/mL	<0.05 EU/mL	<0.05 EU/mL	<0.05 EU/mL
Sterility	Sterile	Sterile	N.T.	N.T.	N.T.	Sterile
UV-VIS absorption peaks	Peaks at 280 ± 3 nm and 775 ± 3 nm.	Conform	Conform	Conform	Conform	Conform
Visible particles	Practically free of particles	Conform	Conform	Conform	Conform	Conform
Subvisible particles	Particles ≥10 µm ≤6000/container Particles ≥25 µm ≤600/container	≥10 µm: 200/vial ≥ 25 µm: 0.7/vial	N.T.	N.T.	N.T.	≥10 µm: 544/vial ≥ 25 µm: 9/vial

Table S6: Stability results of durvalumab-680LT at storage for 12 months at 15-25 °C.

Test	Specification	0	1	3	6	12
Protein monomer concentration	Release: 0.95 – 1.05 mg/mL Shelf-life: 0.90 – 1.10 mg/mL	1.03 ± 0.01 mg/mL	1.02 ± 0.01 mg/mL	1.06 ± 0 mg/mL	1.01 ±0.01 mg/mL	1.04 ± 0.006 mg/mL
Protein aggregates	Release: ≤5.0% Shelf-life: ≤10%	N.D.	N.D.	N.D.	N.D.	N.D.
Unconjugated IRDye 680LT	Release: ≤5.0% Shelf-life: ≤10%	1.3 ± 0.1%	1.6 ± 0.1%	1.6 ± 0.06%	2.1 ± 0.06%	1.9 ± 0.06%
Protein monomer identity	Retention time comparable to reference standard	Conform	Conform	Conform	Conform	Conform
Protein monomer integrity	Peak shape comparable to reference standard; no shoulders, minimal tailing	Conform	Conform	Conform	Conform	Conform
Target binding affinity	EC_{50}ref/EC_{50}tracer 50 – 200%	15.466/15.83 97.7%	14.84/15.82 93.8%	10.97/15.035 73.0%	14715/23.338 63.1%	(14.366/19.948) 72.0%
Appearance (turbidity)	Clear to slightly opalescent solution	Conform	Conform	Conform	Conform	Conform
Appearance (colour)	For information	Conform	Ref 4-5	Ref 5	Ref 5	Ref 5
Container closure and label	Closure intact, label legible and intact.	Conform	Conform	Conform	Conform	Conform
Extractable volume	≥5.0 mL	≥5.0 mL	≥5.0 mL	≥5.0 mL	≥5.0 mL	≥5.0 mL
pH	6.9 – 7.1	6.9	6.9	6.9	6.9	7.0
Osmolality	270 -310 mOsmol/kg	294 mOsmol/kg	291 mOsmol/kg	290 mOsmol/kg	291 mOsmol/kg	293 mOsmol/kg
Residual solvents (DMSO)	≤50.0 mg/L	16 mg/L	N.T.	N.T.	N.T.	N.T.
Bacterial endotoxins	≤5.0 EU/mL	<0.1 EU/mL	<0.1 EU/mL	<0.1 EU/mL	<0.5 EU/mL	<0.5 EU/mL
Sterility	Sterile	Sterile	N.T.	N.T.	Sterile	Sterile
UV-VIS absorption peaks	Peaks at 280 ± 3 nm and 679 ± 3 nm.	Conform	Conform	Conform	Conform	Conform
Visible particles	Practically free of particles	Conform	Conform	Conform	Conform	Conform
Subvisible particles	Particles ≥10 µm ≤6000/container Particles ≥25 µm ≤600/container	≥10 µm: 85 ≥ 25 µm: 3	N.T.	N.T.	N.T.	N.T.

Table S7: Stability results of nivolumab-800CW at storage for 12 months at 15-25 °C.

Test	Specification	0	1	3	6	12
Protein monomer concentration	Release: 0.95 – 1.05 mg/mL Shelf-life: 0.90 – 1.10 mg/mL	0.98 ± 0.003 mg/mL	0.99 ± 0.006 mg/mL	1.00 ± 0.006 mg/mL	0.97 ± 0.006 mg/mL	0.95 ± 0 mg/mL
Protein aggregates	Release: ≤5.0% Shelf-life: ≤10%	N.D.	N.D.	N.D.	N.D.	2.2 ±0.06%
Unconjugated IRDye 800CW	Release: ≤5.0% Shelf-life: ≤10%	0.4 ± 0.09%	0.3 ± 0.1%	1.3 ± 0.06%	1.2 ± 0%	4.3 ±0.06%
Protein monomer identity	Retention time comparable to reference standard	Conform	Conform	Conform	Conform	Conform
Protein monomer integrity	Peak shape comparable to reference standard; no shoulders, minimal tailing	Conform	Conform	Conform	Conform	Conform
Target binding affinity	EC_{50}ref/EC_{50}tracer 50 – 200%	42.554/70.709 60%	40.097/51.706 78%	37.343/63.598 60%	69.634/78.646 89%	39.577/47.281 84%
Appearance (turbidity)	Clear to slightly opalescent solution	Conform	Conform	Conform	Conform	Conform
Appearance (colour)	For information	Ref 5	Ref 5	Ref 5	Ref 5	Ref 5
Container closure and label	Closure intact, label legible and intact.	Conform	Conform	Conform	Conform	Conform
Extractable volume	≥5.0 mL	5.0 mL	5.1 mL	5.1 mL	5.1 mL	5.1 mL
pH	6.9 – 7.1	6.9	6.9	6.9	6.9	6.9
Osmolality	270 -310 mOsmol/kg	291 mOsmol/kg	290 mOsmol/kg	291 mOsmol/kg	290 mOsmol/kg	291 mOsmol/kg
Residual solvents (DMSO)	≤50.0 mg/L	28 mg/L	N.T.	N.T.	N.T.	N.T.
Bacterial endotoxins	≤5.0 EU/mL	<0.05 EU/mL	<0.05 EU/mL	<0.05 EU/mL	<0.05 EU/mL	<0.05 EU/mL
Sterility	Sterile	Sterile	N.T.	N.T.	N.T.	Sterile
UV-VIS absorption peaks	Peaks at 280 ± 3 nm and 775 ± 3 nm.	Conform	Conform	Conform	Conform	Conform
Visible particles	Practically free of particles	Conform	Conform	Conform	Conform	Conform
Subvisible particles	Particles ≥10 µm ≤6000/container Particles ≥25 µm ≤600/container	≥10 µm: 200/vial ≥ 25 µm: 0.7/vial	N.T.	N.T.	N.T.	≥10 µm: 228/vial ≥ 25 µm: 3/vial

Table S8: Summary of toxicity results of male mice: clinical signs, mean body weight, food consumption.

Parameter	G1 (0 mg/kg)	G2 (9 mg/kg)	G3 (90 mg/kg)	G4 (86 µg/kg)	G5 (860 µg/kg)
Clinical Signs (abnormal/total)	0/16	0/10	10/16 (blue tail)	0/10	0/16
Body Weight Day 1 (g)	32.92 ± 1.34	33.40 ± 1.55	33.19 ± 1.61	33.66 ± 1.45	33.35 ± 1.44
Body Weight Day 14 (g)	35.48 ± 1.6	N/A	34.25 ± 2,02	N/A	35.51 ± 1.33
Food Cons. Day 1–2 (g/day)	7.70 ± 1,30	N/A	7.77 ± 1,35	N/A	7.01 ± 0.56
Food Cons. Day 11–14 (g/day)	7.11 ± 1,10	N/A	6.60 ± 0.70	N/A	7.01 ± 0.98

Table S9: Summary of toxicity results of female mice: clinical signs, mean body weight, food consumption.

Parameter	G1 (0 mg/kg)	G2 (9 mg/kg)	G3 (90 mg/kg)	G4 (86 µg/kg)	G5 (860 µg/kg)
Clinical Signs (abnormal/total)	0/16	0/10	7/16 (blue tail)	0/10	0/16
Body Weight Day 1 (g)	26.54 ± 1.26	26.89 ± 1.79	27.02 ± 1.51	26.87 ± 1.03	26.82 ± 1.21
Body Weight Day 14 (g)	27.55 ± 1.18	N/A	28.67 ± 1.26	N/A	28.21 ± 1.14
Food Cons. Day 1–2 (g/day)	7.14 ± 0.63	N/A	6.50 ± 0.52	N/A	6.91 ± 0.58
Food Cons. Day 11–14 (g/day)	5.87 ± 0.47	N/A	6.01 ± 0.49	N/A	5.65 ± 0.94

Table S10: Clinical parameters of male mice.

Group		G1 (0 mg/kg)		G2 (9 mg/kg)	G3 (90 mg/kg)		G4 (86 µg/kg)	G5 (860 µg/kg)	
Day		2	15	2	2	15	2	2	15
		Mean ± SD	Mean ± SD	Mean ± SD	Mean ± SD	Mean ± SD	Mean ± SD	Mean ± SD	Mean ± SD
Weight									
Terminal Fasting BW	g	29.81 ± 1.56	32.73 ± 1.36	30.45 ± 2.03	29.48 ± 2.32	31.93 ± 2.17	30.23 ± 1.93	30.33 ± 2.27	32.84 ± 1.27
Adrenals	g	0.0082 ± 0.0018	0.0069 ± 0.0016	0.0080 ± 0.0016	0.0071 ± 0.0011	0.0076 ± 0.0019	0.0070 ± 0.0019	0.0079 ± 0.0015	0.0071 ± 0.0017
Brain	g	0.5029 ± 0.0260	0.5171 ± 0.0264	0.5097 ± 0.0395	0.05120 ± 0.0181	0.5156 ± 0.0150	0.5217 ± 0.0198	0.5115 ± 0.0388	0.5119 ± 0.0274
Epididymides	g	0.0939 ± 0.0105	0.1018 ± 0.0134	0.0971 ± 0.0120	0.0888 ± 0.0094	0.1135 ± 0.0090	0.0909 ± 0.0111	0.0975 ± 0.0097	0.1062 ± 0.0249
Heart	g	0.1672 ± 0.0108	0.1782 ± 0.0142	0.01705 ± 0.0190	0.1751 ± 0.0172	0.1774 ± 0.0175	0.1689 ± 0.0128	0.1827 ± 0.0131**	0.1744 ± 0.0162
Kidneys	g	0.5142 ± 0.0393	0.5315 ± 0.0245	0.5159 ± 0.0572	0.5239 ± 0.0816	0.5575 ± 0.0639	0.5390 ± 0.0649	0.5263 ± 0.0514	0.5616 ± 0.0627
Liver with gall bladder	g	1.808 ± 0.198	1.7046 ± 0.1822	1.906 ± 0.259	1.687 ± 0.214	1.7903 ± 0.1402	1.795 ± 0.287	1.808 ± 0.239	1.6812 ± 0.1500
Prostate	g	0.0707 ± 0.0125	0.0723 ± 0.0155	0.0600 ± 0.0087	0.0679 ± 0.0126	0.0736 ± 00134	0.0648 ± 0.0127	0.0714 ± 0.0151	0.0720 ± 0.0108
Seminal vesicles & Coagulating glands	g	0.2578 ± 0.0512	0.3100 ± 0.0728	0.2604 ± 0.0391	0.2727 ± 0.522	0.3258 ± 0.0173	0.2759 ± 0.0665	0.2627 ± 0.0389	0.3549 ± 0.0486
Spleen	g	0.1020 ± 0.0167	0.0951 ± 0.0122	0.1014 ± 0.0219	0.0930 ± 0.0150	0.0988 ± 0.0130	0.0996 ± 0.0166	0.0974 ± 0.0220	0.0898 ± 0.0066
Testes	g	0.2183 ± 0.0159	0.2369 ± 0.0447	0.2257 ± 0.0364	0.2204 ± 0.0256	0.2665 ± 0.0235	0.2238 ± 0.0191	0.2267 ± 0.0187	0.2478 ± 0.0390
Thymus	g	0.0455 ± 0.0083	0.0368 ± 0.0061	0.0478 ± 0.0033	0.0422 ± 0.0096	0.0430 ± 0.0102	0.0454 ± 0.0088	0.0473 ± 0.0102	0.0415 ± 0.0097
Haematology									
Red blood cells	10^12/L	9.40 ± 0.55	10.75 ± 1.18	8.66 ± 1.85	9.83 ± 0.43	9.48 ± 0.54	9.51 ± 0.80	9.36 ± 0.41	10.06 ± 0.34
Haemoglobin	g/L	155 ± 8	158 ± 3	142 ± 22	156 ± 6	147 ± 12	158 ± 6	154 ± 5	153 ± 6
Haematocrit	L/L	0.522 ± 0.036	0.587 ± 0.071	0.478 ± 0.096	0.525 ± 0.017	0.504 ± 0.019	0.520 ± 0.029	0.522 ± 0.023	0.525 ± 0.030
MCV	fL	55.6 ± 2.0	54.6 ± 0.6	55.4 ± 2.0	53.4 ± 2.1	53.2 ± 1.4	54.8 ± 2.3	55.8 ± 1.1	52.1 ± 1.3 *
MCH	pg	16.50.8	14.8 ± 1.7	16.7 ± 1.6	15.9 ± 0.6	15.5 ± 0.8	16.7 ± 11	16.5 ± 0.6	15.2 ± 0.1
MCHC	g/L	296 ± 8	271 ± 34	301 ± 24	298 ± 4	291 ± 14	305 ± 16	297 ± 9	291 ± 6
Retic A	10^12/L	0.299 ± 0.056	0.321 ± 0.053	0.284 ± 0.070	0.281 ± 0.052	0.297 ± 0.020	0.294 ± 0.031	0.240 ± 0.030	0.332 ± 0.029
Retic	%	3.17 ± 0.49	2.98 ± 0.36	3.25 ± 0.21	2.85 ± 0.46	3.14 ± 0.04	3.11 ± 0.40	2.56 ± 0.24	3.30 ± 0.20
Platelet count	10^9/L	1481 ± 80	1456 ± 84	1396 ± 357	1485 ± 66	1319 ± 250	1355 ± 266	1478 ± 218	1455 ± 98
White blood cells	10^9/L	3.85 ± 1.79	7.12 ± 2.70	4.98 ± 2.32	4.48 ± 1.54	3.97 ± 1.08	5.15 ± 1.66	3.13 ± 0.87	4.50 ± 2.19
Neutrophils A	10^9/L	0.54 ± 0.22	0.78 ± 0.24	0.70 ± 0.31	0.93 ± 0.53	0.53 ± 0.22	0.69 ± 0.10	0.64 ± 0.17	0.49 ± 0.17
Lymphocytes A	10^9/L	3.12 ± 1.44	6.00 ± 2.33	4.05 ± 1.93	3034 ± 1.10	3.28 ± 0.89	4.22 ± 1.52	2.29 ± 0.72	3.80 ± 1.95
Monocytes A	10^9/L	0.07 ± 0.06	0.12 ± 0.03	0.09 ± 0.05	0.08 ± 0.03	0.09 ± 0.03	0.09 ± 0.03	0.06 ± 0.02	0.07 ± 0.05
Basophils A	10^9/L	0.01 ± 0.01	0.03 ± 0.02	0.03 ± 0.02	0.02 ± 0.01	0.01 ± 0.00*	0.02 ± 0.01	0.01 ± 0.01	0.01 ± 0.02
Eosinophils A	10^9/L	0.09 ± 0.07	0.15 ± 0.08	0.09 ± 0.04	0.06 ± 0.06	0.07 ± 0.03	0.10 ± 0.03	0.11 ± 0.06	0.10 ± 0.04

Prothrombin Time	Sec	11.9 ± 1.7	14.0 ± 0.9	12.1 ± 1.1	10.4 ± 0.9	12.5 ± 0.4	11.1 ± 0.9	11.4 ± 1.2	12.2 ± 1.6
APTT	Sec	13.2 ± 2.8	13.6 ± 2.3	18.8 ± 2.5**	16.6 ± 4.1	15.9 ± 3.2	16.9 ± 3.8	16.1 ± 4.9	17.0 ± 4.1
Clinical Chemistry									
Glucose	Mmol/L	11.56 ± 1.12	12.51 ± 1.86	11.60 ± 1.16	10.84 ± 2.21	12.52 ± 2.04	10.95 ± 1.88	11.64 ± 3.10	12.12 ± 0.74
Blood urea nitrogen	Mmol/L	7.50 ± 3.01	6.05 ± 0.51	5.97 ± 0.63	6.60 ± 1.98	6.30 ± 0.18	5.54 ± 0.554	6.53 ± 1.56	6.73 ± 1.08
Creatine	µmol/L	15(n = 1)	21 ± 6	20 ± 4	16 ± 1	22 ± 1	13 (n = 1)	15 ± 2	22 ± 1
AST	U/L	64 ± 17	59 ± 7	76 ± 17	67 ± 11	64 ± 3	61 ± 12	67 ± 26	63 ± 16
ALT	U/L	50 ± 21	43 ± 11	43 ± 5	52 ± 23	39 ± 6	47 ± 14	57 ± 45	36 ± 9
GGT	U/L	2 ± 1	4 ± 1	2 ± 0	2 ± 0	3 ± 1	3 ± 1	2 ± 1	3 ± 1
ALP	U/L	149 ± 19	85 ± 27	108 ± 16*	130 ± 22	98 ± 22	123 ± 29	102 ± 20*	133 ± 26
LDH	U/L	259 ± 48	211 ± 88	363 ± 258	258 ± 37	141 ± 6	260 ± 78	252 ± 139	249 ± 9
T. Bilirubin	µmol/L	2.33 ± 1.25	3.25 ± 0.93	2.40 ± 0.21	2.46 ± 0.63	4.08 ± 0.52	1.89 ± 0.38	2.45 ± 0.81	2.81 ± 0.41
T. Cholesterol	Mmol/L	3.92 ± 0.64	4.50 ± 0.787	4.37 ± 0.71	4.02 ± 0.58	4.04 ± 0.20	4.24 ± 0.42	3.91 ± 0.27	4.37 ± 0.19
Triglycerides	Mmol/L	1.01 ± 0.31	0.93 ± 0.41	.01 ± 0.20	0.67 ± 0.29	0.71 ± 0.25	0.71 ± 0.38	0.76 ± 0.19	0.77 ± 0.16
T. protein	g/L	53.5 ± 4.6	50.8 ± 2.7	53.3 ± 1.3	54.1 ± 2.0	50.2 ± 1.6	52.6 ± 0.8	53.9 ± 3.7	51.6 ± 3.3
ALB	g/L	27.5 ± 4.2	23.8 ± 1.9	26.4 ± 3.1	28.1 ± 1.8	24.0 ± 0.6	27.3 ± 1.7	27.4 ± 1.4	24.9 ± 2.3
GLOB	g/L	26.1 ± 2.8	27.0 ± 1.1	26.9 ± 2.8	26.0 ± 1.7	26.2 ± 1.1	25.3 ± 1.4	26.4 ± 2.4	26.7 ± 1.0
Albumin/Globulin	ratio	1.06 ± 0.21	0.88 ± 0.06	1.00 ± 0.19	1.08 ± 0.12	0.92 ± 0.03	1.08 ± 0.12	1.04 ± 0.05	0.93 ± 0.05
PI	Mmol/L	2.54 ± 0.43	2.49 ± 0.26	2.59 ± 0.36	2.53 ± 0.32	2.25 ± 0.16	2.62 ± 0.17	2.78 ± 0.17	2.51 ± 0.33
Calcium	mEq/L	2.40 ± 0.20	2.40 ± 0.13	2.47 ± 0.07	2.43 ± 0.06	2.30 ± 0.03	2.43 ± 0.17	2.40 ± 0.12	2.48 ± 0.07
Sodium	mEq/L	156.6 ± 4.4	154.0 ± 1.3	155.5 ± 4.9	152.4 ± 2.5	154.6 ± 1.3	155.0 ± 2.9	157.3 ± 1.3	154.1 ± 0.7
Potassium	mEq/L	4.11 ± 0.23	3.97 ± 0.314	4.06 ± 0.29	3.84 ± 0.34	3.57 ± 0.40	3.83 ± 0.34	3.64 ± 0.26	4.06 ± 0.13
Chlorine	mEq/L	113.3 ± 3.0	115.3 ± 1.2	113.3 ± 3.9	110.4 ± 2.0	112.4 ± 2.2	112.0 ± 2.1	114.1 ± 1.3	110.9 ± 0.4 *

*: Significantly lower than the vehicle control group at $p < 0.05$ at the same timepoint
**: Significantly higher than the vehicle control group at $p < 0.05$ at the same timepoint

Table S11: Clinical parameters of female mice.

Group		G1 (0 mg/kg)		G2 (9 mg/kg)	G3 (90 mg/kg)		G4 (86 µg/kg)	G5 (860 µg/kg)	
Day		2	15	2	2	15	2	2	15
		Mean ± SD	Mean ± SD	Mean ± SD	Mean ± SD	Mean ± SD	Mean ± SD	Mean ± SD	Mean ± SD
Weight									
Terminal Fasting BW	g	24.33 ± 1.52	25.20 ± 0.90	24.01 ± 2.11	23.87 ± 1.96	26.15 ± 1.41	24.39 ± 1.36	23.97 ± 1.41	25.57 ± 0.97
Adrenals	g	0.011 ± 0.0015	0.0108 ± 0.0011	0.0100 ± 0.0017	0.0105 ± 0.0020	0.0111 ± 0.0023	0.0100 ± 0.0015	0.0116 ± 0.0015	0.0122 ± 0.0019
Brain	g	0.4950 ± 0.0270	0.5049 ± 0.0339	0.5146 ± 0.0315	0.5026 ± 0.0222	0.5186 ± 0.0190	0.4974 ± 0.0290	0.5107 ± 0.0271	0.4998 ± 0.0160
Heart	g	0.1438 ± 0.0108	0.1445 ± 0.0126	0.1487 ± 0.0103	0.1425 ± 0.0126	0.1472 ± 0.0086	0.1403 ± 0.0128	0.1449 ± 0.0159	0.1432 ± 0.0116
Kidneys	g	0.3362 ± 0.0152	0.3684 ± 0.0187	0.3440 ± 0.0360	0.3397 ± 0.0435	0.3612 ± 0.0293	0.3472 ± 0.0344	0.3367 ± 0.0328	0.3365 ± 0.0382
Liver with gall bladder	g	1.4351 ± 0.1723	1.2917 ± 0.0729	1.3429 ± 0.2269	1.3763 ± 0.2392	1.3769 ± 0.1567	1.2736 ± 0.1032*	1.3107 ± 0.1249	1.2488 ± 0.1113
Ovaries	g	0.0267 ± 0.0070	0.0244 ± 0.0072	0.0248 ± 0.0044	0.0305 ± 0.0073	0.0259 ± 0.0045	0.0269 ± 0.0053	0.0284 ± 0.0057	0.0290 ± 0.0073
Spleen	g	0.1004 ± 0.0154	0.1021 ± 0.0221	0.0991 ± 0.0103	0.1104 ± 0.0462	0.1129 ± 0.0163	0.0954 ± 0.0196	0.1074 ± 0.0195	0.1169 ± 0.0270
Thymus	g	0.0574 ± 0.0155	0.0524 ± 0.0113	0.0589 ± 0.0117	0.0504 ± 0.0083	0.0547 ± 0.0116	0.0584 ± 0.0160	0.0641 ± 0.0141	0.0560 ± 0.0159
Uterus with cervix	g	0.2492 ± 0.0731	0.2181 ± 0.0812	0.2237 ± 0.1067	0.2558 ± 0.1014	0.2007 ± 0.0637	0.2944 ± 0.1330	0.2267 ± 0.1043	0.2543 ± 0.0800
Haematology									
Red blood cells	10^12/L	9.86 ± 0.42	9.92 ± 0.07	9.63 ± 0.49	9.50 ± 0.50	9.39 ± 0.54	9.70 ± 0.38	9.44 ± 0.39	9.49 ± 0.23*
Haemoglobin	g/L	159 ± 7	161 ± 4	157 ± 6	156 ± 6	154 ± 7	159 ± 4	154 ± 8	149 ± 5*
Haematocrit	L/L	0.538 ± 0.025	0.533 ± 0.002	0.522 ± 0.017	0.524 ± 0.011	0.520 ± 0.012	0.515 ± 0.017	0.510 ± 0.023	0.515 ± 0.011*
MCV	fL	54.6 ± 1.1	53.8 ± 0.6	54.3 ± 2.9	55.3 ± 1.9	55.4 ± 2.1	53.1 ± 1.7	54.1 ± 1.5	54.3 ± 0.4
MCH	pg	16.2 ± 1.1	16.3 ± 0.5	16.3 ± 0.8	16.4 ± 0.5	16.4 ± 0.3	16.4 ± 0.4	16.3 ± 0.3	15.8 ± 0.1
MCHC	g/L	297 ± 22	303 ± 6	301 ± 6	297 ± 8	296 ± 7	309 ± 4	301 ± 9	290 ± 3*
Retic A	10^12/L	0.337 ± 0.050	0.251 ± 0.032	0.278 ± 0.025	0.296 ± 0.025	0.312 ± 0.019**	0.282 ± 0.058	0.276 ± 0.43	0.333 ± 0.029
Retic	%	3.41 ± 0.38	2.53 ± 0.31	2.89 ± 0.26	3.12 ± 0.31	3.34 ± 0.34**	2.90 ± 0.56	2.93 ± 0.43	3.52 ± 0.39**
Platelet count	10^9/L	1338 ± 144	1332 ± 122	1200 ± 170	1356 ± 125	1183 ± 47	1211 ± 85	1363 ± 73	1235 ± 165
White blood cells	10^9/L	7.50 ± 2.38	4.79 ± 1.11	4.98 ± 1.45	4.99 ± 1.95	5.19 ± 2.33	5.78 ± 3.25	5.90 ± 1.75	3.81 ± 2.25
Neutrophils A	10^9/L	0.84 ± 0.43	0.43 ± 0.07	0.74 ± 0.31	0.74 ± 0.41	0.69 ± 0.30	0.77 ± 0.54	0.75 ± 0.24	0.45 ± 0.21
Lymphocytes A	10^9/L	6.24 ± 1.87	4.16 ± 1.07	3.94 ± 1.09	3.98 ± 1.59	4.21 ± 1.93	4.69 ± 2.57	4.86 ± 1.49	3.19 ± 1.93
Monocytes A	10^9/L	0.12 ± 0.09	0.03 ± 0.02	0.09 ± 0.04	0.06 ± 0.02	0.04 ± 0.02	0.08 ± 0.07	0.07 ± 0.02	0.04 ± 0.03
Basophils A	10^9/L	0.05 ± 0.02	0.05 ± 0.02	0.03 ± 0.02	0.02 ± 0.02	0.03 ± 0.03	0.03 ± 0.04	0.05 ± 0.03	0.03 ± 0.02
Eosinophils A	10^9/L	0.20 ± 0.08	0.09 ± 0.06	0.15 ± 0.06	0.15 ± 0.06	0.19 ± 0.11	0.17 ± 0.13	0.15 ± 0.04	0.08 ± 0.05
Prothrombin Time	Sec	12.1 ± 0.8	12.0 ± 1.2	10.7 ± 0.5 *	11.4 ± 1.5	14.3 ± 2.4	11.4 ± 1.2	10.7 ± 0.4	13.1 ± 1.4
APTT	Sec	12.5 ± 4.2	15.8 ± 4.2	15.8 ± 4.4	14.1 ± 4.6	20.4 ± 3.6	19.1 ± 5.4	18.2 ± 3.1	20.5 ± 2.0
Clinical Chemistry									
Glucose	Mmol/L	9.29 ± 0.85	9.74 ± 0.92	9.64 ± 1.58	9.15 ± 1.03	9.43 ± 2.45	9.28 ± 1.16	9.52 ± 1.08	10.43 ± 0.33

Blood urea nitrogen	Mmol/L	6.59 ± 3.82	4.18 ± 1.32	5.06 ± 0.58	4.14 ± 0.61*	5.37 ± 0.21	4.70 ± 0.67	5.57 ± 3.57	4.83 ± 0.45
Creatine	μmol/L	13 (n = 1)	19 ± 1	18 (n = 1)	16 (n = 1)	20 ± 2	-LLOQ	-	18 ± 2
AST	U/L	76 ± 31	59 ± 18	95 ± 40	92 ± 23	64 ± 9	76 ± 18	79 ± 25	88 ± 13
ALT	U/L	42 ± 19	28 ± 3	44 ± +9	41 ± 18	32 ± 1	33 ± 12	42 ± 22	27 ± 15
GGT	U/L	2 ± 1	3 ± 1	1 ± 1	2 ± 1	4 ± 1	2 ± 0	1 ± 2	3 ± 1
ALP	U/L	137 ± 39	116 ± 16	133 ± 33	145 ± 38	127 ± 27	134 ± 24	154 ± 47	147 ± 38
LDH	U/L	255 ± 71	175 ± 29	304 ± 79	274 ± 125	183 ± 21	239 ± 45	292 ± 95	216 ± 28
T. Bilirubin	μmol/L	1.54 ± 0.73	2.54 ± 0.53	1.60 ± 0.46	2.27 ± 0.48	2.43 ± 0.29	1.55 ± 0.58	1.77 ± 0.40	2.02 ± 1.11
T. Cholesterol	Mmol/L	3.23 ± 0.19	3.00 ± 0.09	3.27 ± 0.59	2.98 ± 0.55	3.35 ± 0.28	3.56 ± 0.83	3.19 ± 0.45	2.89 ± 0.9
Triglycerides	Mmol/L	0.99 ± 0.62	0.53 ± 0.04	0.67 ± 0.19	0.51 ± 0.19	0.62 ± 0.08	0.67 ± 0.17	0.60 ± 0.19	0.40 ± 0.07
T. protein	g/L	52.2 ± 2.7	54.0 ± 0.2	53.1 ± 2.0	53.9 ± 2.9	51.5 ± 3.1	52.5 ± 2.0	53.0 ± 2.4	54.9 ± 3.5
ALB	g/L	30.1 ± 1.7	30.6 ± 2.6	29.4 ± 2.0	30.2 ± 2.9	27.1 ± 1.9	29.6 ± 1.8	30.2 ± 1.7	30.0 ± 1.6
GLOB	g/L	22.2 ± 1.2	23.4 ± 2.5	23.7 ± 1.8	23.6 ± 3.3	24.4 ± 1.4	23.0 ± 0.5	22.8 ± 0.8	25.0 ± 2.0
Albumin/Globulin	ratio	1.36 ± 0.05	1.32 ± 0.27	1.25 ± 0.15	1.31 ± 0.29	.11 ± 0.04	1.29 ± 0.08	1.2 ± 0.04	1.20 ± 0.04
Pi	Mmol/L	2.50 ± 0.29	2.83 ± 0.46	2.47 ± 0.27	2.31 ± 0.19	2.88 ± 0.66	2.57 ± 0.15	2.86 ± 0.14 **	2.73 ± 0.10
Calcium	mEq/L	2.48 ± 0.28	2.55 ± 0.06	2.58 ± 0.15	2.8 ± 0.12	2.55 ± 0.07	2.50 ± 0.05	2.59 ± 0.32	2.47 ± 0.07
Sodium	mEq/L	155.1 ± 1.8	154.3 ± 0.5	153.2 ± 3.6	151.5 ± 4.2	154.8 ± 1.2	151.9 ± 4.3	153.7 ± 3.0	155.5 ± 0.8
Potassium	mEq/L	3.88 ± 0.32	3.70 ± 0.49	3.92 ± 0.36	3.86 ± 0.19	3.63 ± 0.69	3.72 ± 0.21	3.90 ± 0.30	3.78 ± 0.09
Chlorine	mEq/L	113.6 ± 1.7	113.3 ± 0.6	111.5 ± 2.8	112.0 ± 4.6	112.7 ± 0.5	111.1 ± 3.9	113.7 ± 3.4	114.0 ± 1.1

*: Significantly lower than the vehicle control group at $p < 0.05$ at the same timepoint

**: Significantly higher than the vehicle control group at $p < 0.05$ at the same timepoint

Table S12: Stability testing, timepoints, tests and both release and shelf-life specifications. Tests were performed up until 12 months for 15-25 °C. Tests will be performed up until 24 months for 2-8 °C.

		Time after production (months)							
Test	Specification	0	1	2	3	6	12	18	24
Protein monomer concentration	Release: 0.95 – 1.05 mg/mL<br>Shelf-life: 0.90 – 1.10 mg/mL	X	X	X	X	X	X	X	X
Protein aggregates	Release: ≤5.0%<br>Shelf-life: ≤10%	X	X	X	X	X	X	X	X
Unconjugated IRDye 800CW/ IRDye 680LT	Release: ≤5.0%<br>Shelf-life: ≤10%	X	X	X	X	X	X	X	X
Protein monomer identity	Retention time comparable to reference standard	X	X	X	X	X	X	X	X
Protein monomer integrity	Peak shape comparable to reference standard; no shoulders, minimal tailing	X	X	X	X	X	X	X	X
Target binding affinity	50 – 200%	X	X	X	X	X	X	X	X
Appearance (turbidity)	Clear to slightly opalescent solution	X	X	X	X	X	X	X	X
Appearance (colour)	For information	X	X	X	X	X	X	X	X
Container closure and label	Closure intact, label legible and intact.	X	X	X	X	X	X	X	X
Extractable volume	≥5.0 mL	X	X	X	X	X	X	X	X
pH	Release: 6.9–7.1<br>Shelf-life: 6.9–7.1	X	X	X	X	X	X	X	X
Osmolality	270 – 310 mOsmol/kg	X	X	X	X	X	X	X	X
Residual solvents (DMSO)	≤50.0 mg/L	X							
Bacterial endotoxins	≤5.0 EU/mL	X					X		X
Sterility	Sterile	X					X		X
UV-VIS absorption peaks	Peaks at 280 ± 3 nm and 679 ± 3 nm or 775 ± 3 for 680LT and 800CW, respectively.	X	X	X	X	X	X	X	X
Visible particles	Practically free of particles	X	X	X	X	X	X	X	X
Subvisible particles	Particles ≥10 µm ≤6000/container<br>Particles ≥25 µm ≤600/container	X					X		X

Figure S1: Stability results of the buffer panel of durvalumab-680LT at 15-25 °C. Release specifications are displayed with dotted lines, and end of shelf-life specifications are displayed with dashed lines. (A) Protein concentration, (B) percentage of free dye and (C) percentage of aggregates. See supplementary table 1 for all used buffers.

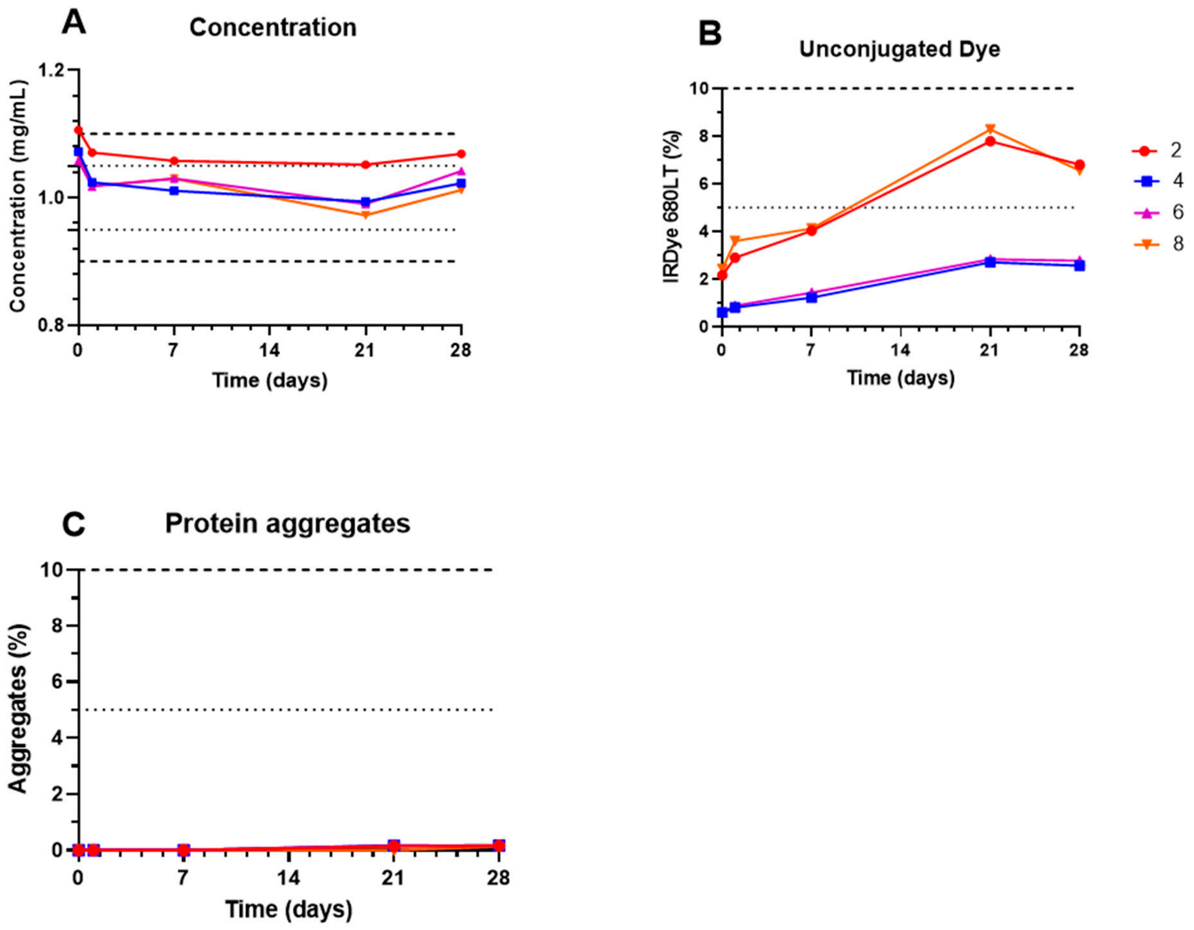

Figure S2: Stability results of the buffer panel of nivolumab-800CW at 15-25 °C. Release specifications are displayed with dotted lines, and end of shelf-life specifications are displayed with dashed lines. (A) Protein concentration, (B) percentage of free dye and (C) percentage of aggregates. See supplementary table 1 for all used buffers.

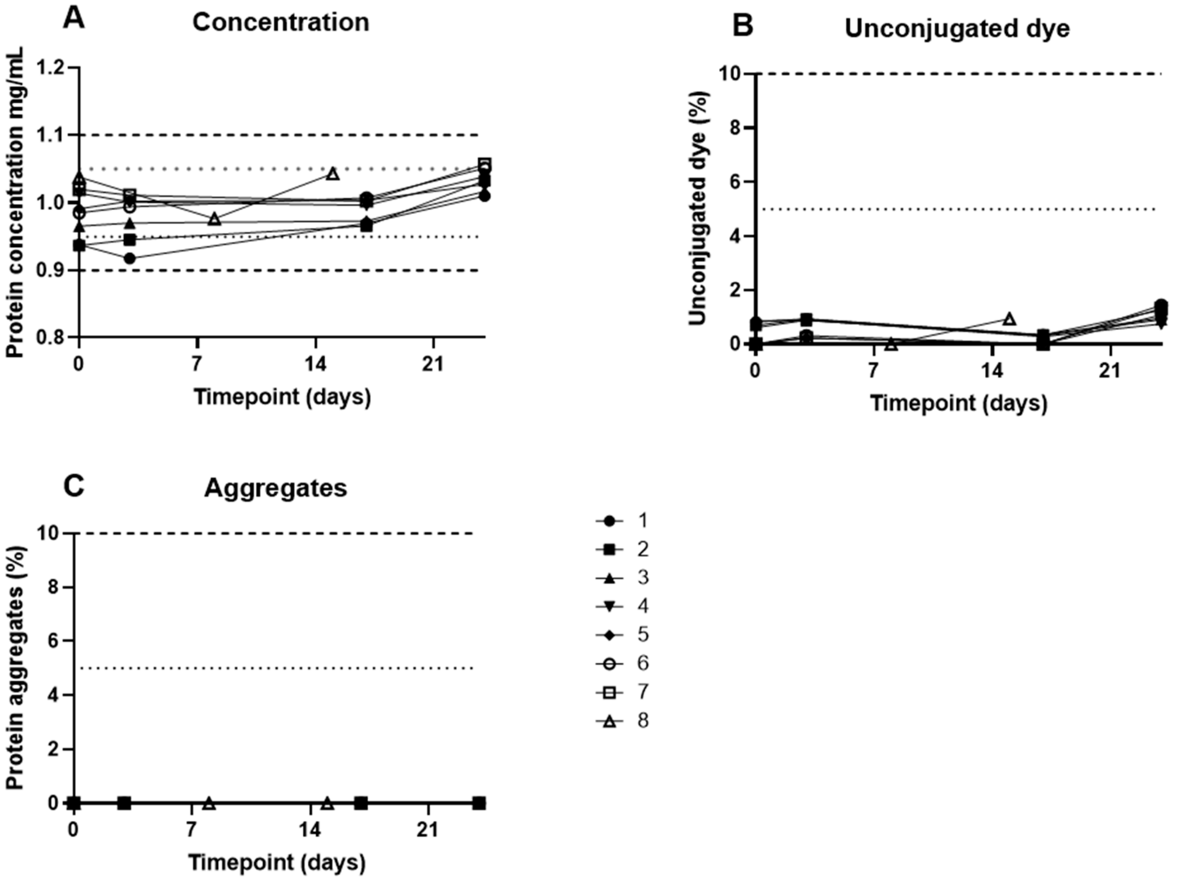

Figure S3: Stability results of both durvalumab-680LT and nivolumab-800CW at 15-25 °C. Release specifications are displayed with dotted lines, and end of shelf-life specifications are displayed with dashed lines. (A) Protein concentration, (B) percentage of free dye, (C) percentage of aggregates, and (D) target binding affinity of both tracers. A-C are means ± standard deviation of three different measurements, in D mean is reported of two measurements.

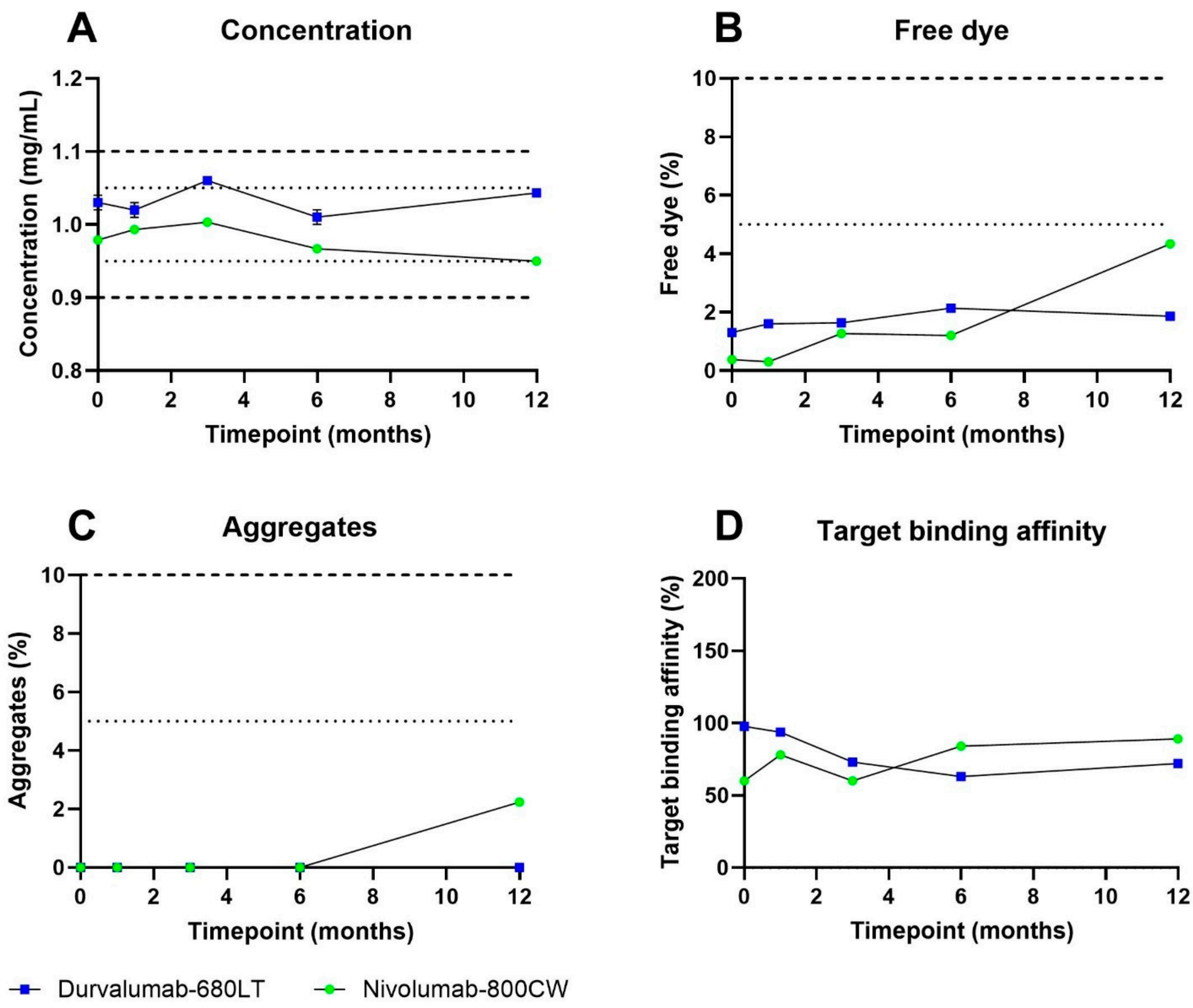

Supplement: Supplementary file 1 [file pharmaceuticals-18-01501-s001.zip › pharmaceuticals-3899461-supplementary.pdf]
